# Experimental infection of wild boars (*Sus scrofa*) with *Rickettsia rickettsii* and evaluation of the transmission potential to *Amblyomma sculptum* ticks

**DOI:** 10.1186/s13071-024-06612-y

**Published:** 2025-01-16

**Authors:** Lucianne Cardoso Neves, Lina de Campos Binder, Warley Vieira de Freitas Paula, Nicolas Jalowitzki de Lima, Ennya Rafaella Neves Cardoso, Rayane Almeida Santos, Raphaela Bueno Mendes Bittencourt, Gracielle Teles Pádua, Gabriel Cândido dos Santos, Mariana Avelar Tavares, Maria Carolina de Azevedo Serpa, Adriano Pinter, Artur Luiz de Almeida Felicio, Marcelo B. Labruna, Felipe da Silva Krawczak

**Affiliations:** 1 Veterinary and Animal Science School, Federal University of Goiás, Goiânia, Goiás 74690-900 Brazil; 2 Department of Preventive Veterinary Medicine and Animal Health, School of Veterinary Medicine and Animal Science, University of São Paulo, São Paulo, 05508-270 Brazil; 3 Coordination of Agricultural Defense, Secretary of Agriculture and Supply, Government of the State of São Paulo, São Paulo, Brazil

**Keywords:** Tick-borne diseases, Brazilian spotted fever, Suidae, *Amblyomma sculptum*

## Abstract

**Background:**

Brazilian spotted fever is a tick-borne disease caused by the bacterium *Rickettsia rickettsii*, whose main vector in Brazil is the tick *Amblyomma sculptum*. Amplifying hosts are essential for the perpetuation of this bacterium in the tick population as they can be sources of infection during bacteremic periods. Recent studies demonstrated the ability of suids (*Sus scrofa*) to sustain populations of *A. sculptum*, one of the main tick species found parasitizing wild boars in the midwestern and southeastern regions of Brazil. In this study, wild boars were experimentally infected with *R. rickettsii* by tick infestation and were evaluated for their ability to transmit the infection to *A. sculptum* ticks, under laboratory conditions.

**Methods:**

Four wild boars were infected with *R. rickettsii* through infestation with *R. rickettsii*-infected *A. sculptum* adults (infected group); a fifth wild boar was infested with uninfected *A. sculptum* adults (control group). Simultaneously, the animals were infested with uninfected larvae and nymphs of *A. sculptum*. The wild boars were monitored for 28 days by clinical examination and hematological tests, real-time quantitative PCR (qPCR) of blood for the detection of *Rickettsia* and inoculation of blood in guinea pigs. IgG antibody titers were followed until the end of the experiment. Unfed nymphs and adults, molted from engorged larvae and nymphs that fed on wild boars, were used to infest susceptible guinea pigs and rabbits; some of these unfed ticks were tested by qPCR for rickettsial detection.

**Results:**

The wild boars showed no clinical or hematological alterations, and bacteremia was not detected by qPCR or inoculation of wild boar blood into guinea pigs. Furthermore, wild boars showed a moderate humoral response, with anti-*R. rickettsii* endpoint titers up to 256 or 512. Rickettsial DNA was not detected in molted ticks after acquisition feeding on wild boars. Moreover, no disease or seroconversion was observed in guinea pigs and rabbits that were infested with ticks originated from wild boar acquisition feeding.

**Conclusions:**

Wild boars seroconverted to *Rickettsia* spp. after being infested with *R. rickettsii*-infected *A. sculptum*; however, they did not develop bacteremia and did not act as competent amplifying hosts of *R. rickettsii* for *A. sculptum* ticks.

**Graphical Abstract:**

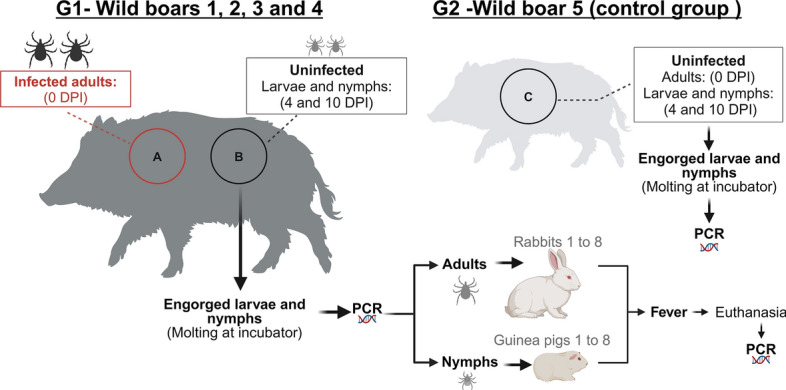

## Background

Bacteria of the genus *Rickettsia* are causative agents of rickettsioses, which are vector-borne zoonoses of extreme relevance for health [1, 2], distributed across several continents, including South America [2]. In Brazil, *Rickettsia rickettsii* is the etiological agent of Brazilian spotted fever (BSF), one of the most lethal tick-borne disease affecting humans worldwide, with a fatality rate of up to 80% in some areas where diagnostic suspicion and specific antibiotic therapy are carried out late [3–5]. Ticks of the species *Amblyomma sculptum* (*Amblyomma cajennense* complex) are the main vectors of *R. rickettsii* in Brazil [6–8], where horses and capybaras are their preferred hosts, contributing to the maintenance of populations of this tick in the environment [7, 9].

In addition to being pathogenic to humans, *R. rickettsii* induces greater mortality of immature stages and reduces the reproduction rate of *A. sculptum* females. In addition, fewer than 50% of infected females transmit *R. rickettsii* transovarially [10, 11]. Therefore, among BSF-endemic areas, only 0.05% to 1% *R. rickettsii-*infected ticks have been reported in the *A. sculptum* populations [6, 12–14]. This condition indicates that an *A. sculptum* population is not capable of sustaining an infection by *R. rickettsii* during successive generations without the participation of vertebrate amplifying hosts, i.e. hosts that once primarily infected, will develop bacteremia for some period of time when new cohorts of infected ticks are created [7, 15]. In this sense, capybaras play an important role in the epidemiology of BSF, as they act as *R. rickettsii* amplifying hosts for the *A. sculptum* tick in Brazil [16–18].

Recent studies have demonstrated the ability of domestic, wild or exotic suidae (*Sus scrofa*) to sustain populations of *A. sculptum* [19, 20], which is one of the main tick species found parasitizing wild boars in the midwestern and southeastern regions of Brazil [21–24]. Wild boars are classified by Brazilian law as an exotic, invasive species, originating from Eurasian wild boars and their hybrids, with hunting nationwide permitted by Normative Instruction No. 03/2013 as a population control and eradication strategy [25]. As a large mammal, wild boars can invade natural and anthropic areas, competing for resources with native wildlife, with the potential to contribute to the maintenance of the life cycle of ticks and tick-borne agents [26–28].

Wild boars are indicated as hosts and reservoirs of several agents of zoonotic diseases in Brazil, including being associated as potential participants in the cycle of rickettsial diseases, such as BSF [22–24, 26, 27]. Previous studies have detected the presence of antibodies against *Rickettsia* spp. antigens of the spotted fever group (SFG) in wild boars [21–23]. These data, together with other studies, suggest that specific activities such as hunting or controlling wild boars, farming and ecotourism in areas inhabited by these animals may increase the risk of human exposure to SFG rickettsiae [22, 26, 27].

Despite the lack of information regarding the ability of wild boar to infect ticks and their role in the transmission cycle of *Rickettsia* spp., incursion into tick habitats may lead to the potential risk of infection, transmission and spread of *Rickettsia* spp. and other tick-borne diseases [22, 24, 26, 27]. In ecosystems where there is an overlapping distribution of wild boars and capybaras in Brazil, for example, this could lead to the synergistic spread of *A. sculptum* and contribute to the risk of an increase in the number of cases of BSF [22, 27]. This highlights the need to elucidate the role of wild boars in the cycle of rickettsioses, especially BSF.

Therefore, the present study experimentally infected wild boars (*S. scrofa*) with *R. rickettsii* and evaluated the role of these animals as an amplifying host for *A. sculptum* ticks, under laboratory conditions.

## Methods

### Animals

Five wild boars (no. 1–5) were obtained from two different BSF-nonendemic areas in São Paulo state, southeastern, Brazil. These animals were born in captivity in legalized breeding farms and consisted of two castrated males (nos. 1 and 2) from a breeding farm in Pirassununga municipality and three females (nos. 3, 4 and 5) from a breeding farm in Piedade municipality. The animals were between 2 and 3 months old and weighed between 13 and 20 kg. They were transported to the Animal Research Facility of the Instituto Pasteur of Mogi Guaçu, São Paulo state, Brazil, where they were kept in individual boxes (3 m × 3 m) and fed daily with a commercial pig pellet diet, corn grains and water ad libitum. For environmental enrichment, wild boars had permanent access to a hay bed. All animals were inspected for ticks; up to the time of the experiment, no ticks were seen.

Laboratory guinea pigs (Hartley strain) and white New Zealand rabbits were purchased from a commercial breeder and housed in standardized laboratory cages. These animals were fed with a commercial guinea pig and rabbit pellet diet, had water ad libitum and were kept in facilities with temperature, photoperiod (12/12 h) and ventilation control. All guinea pigs and rabbits were tick-naïve and bred under standard laboratory sanitary conditions.

All animal procedures were authorized by the Ethics Committee of Animal Use of the University of São Paulo (project no. 8651010623) and Ethics Committee of Animal Use of the Federal University of Goiás (project no. 076/21). All methods were performed in accordance with the guidelines and regulations of the Brazilian National Council of Animal Experimentation (CONCEA).

### *Rickettsia rickettsii*

*Rickettsia rickettsii* strain Itu, originally isolated from the tick *A. sculptum* in Brazil, was used in this study [6]. For the present study, we used the stock derived from a fourth passage of strain Itu that has been maintained by consecutive passages only in guinea pigs, with no in vitro passages [10]. This strain was used to form the *R. rickettsii*-infected *A. sculptum* colony, as described below.

### *Amblyomma sculptum* ticks

Ticks used in the present study derived from a tick colony that was established in the laboratory from unfed adults collected from the environment by flannel cloth dragging and visual search [29] in the experimental farm of the School of Veterinary and Animal Science of the Federal University of Goiás (EVZ/UFG), namely *A. sculptum* strain GYN [30]. This experimental farm (16° 35′ 42″ S, 49° 16′ 50″ W) is located in the municipality of Goiânia, Goiás state, an area non-endemic for BSF. The ticks were identified according to taxonomic key [31, 32]. To confirm that the colony was not naturally infected by SFG rickettsiae, rabbits and guinea pigs used for feeding ticks were tested for the presence of *R. rickettsii*-reactive antibodies before and 21 days after infestation with the ticks (serological protocol described below); none of the rabbits and guinea pigs seroconverted to *R. rickettsii*. Furthermore, molecular analysis (protocol describe below) failed to detect rickettsial DNA in tick samples (egg pools, nymphs and adults) during the establishment of the tick colony.

The *A. sculptum* colony was used to form two separated cohorts in the laboratory, one uninfected (as it was originally) and one infected by *R. rickettsii*. For the latter, the larval and nymphal stages of a single generation were allowed to feed on rickettsemic guinea pigs that were intraperitoneally inoculated with a homogenate of *R. rickettsii* strain Itu-infected guinea pig organs, as previously described [11]. Molecular analysis showed the resultant adult ticks of this generation harbored a 30% infection rate by *R. rickettsii*, based on the testing of 20 unfed individual adults by PCR (protocol described below).

### Experimental infection of wild boars

Right before the first tick infestation on wild boars, the five animals were clinically evaluated and tested by indirect immunofluorescence assay (IFA) for the presence of IgG antibodies that could react to crude antigens of *R. rickettsii* (strain Pampulha) [13], *Rickettsia parkeri* (strain At 24) [33], *Rickettsia amblyommatis* (strain Ac 37) [34] and *Rickettsia bellii* (strain Mogi) [35], as previously described [36, 37].

Wild boars were randomly assigned to form two experimental groups: four animals (no. 1–4) for the infected group (G1) and one (no. 5) for the control group (non-infected group) (G2). Two separated cotton sleeves (15-cm-diameter tick-feeding chambers) were glued on the shaved dorsum of each of the four wild boars of G1, while a single feeding chamber was glued on the dorsum of the G2 wild boar, as previously described [16, 17]. In G1 wild boars, feeding chambers were labeled as chamber A (cranial position on the animal dorsum) and chamber B (caudal position on the animal). The minimum distance between the two chambers was 10 cm.

**Fig. 1:**
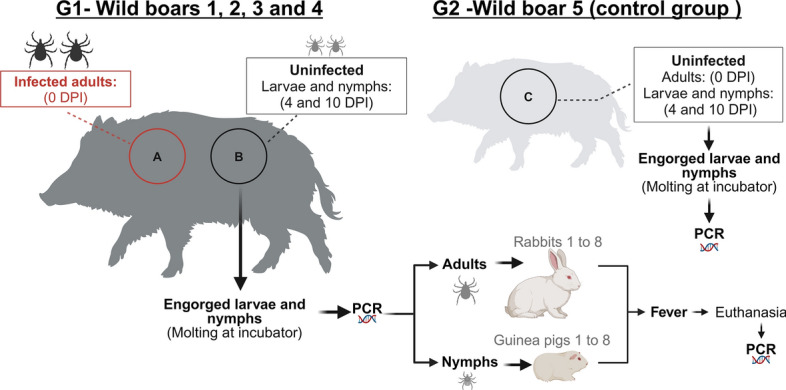
Scheme of experimental procedures of the present study. On 0 day, four wild boars (Group 1) were exposed to rickettsial infection through infestation with *Rickettsia rickettsii*-infected *Amblyomma sculptum* adults, and one other wild boar (Group 2) was infested with uninfected *A. sculptum* adults. The five wild boars were infested with uninfected *A. sculptum* ticks (larvae and nymphs) on 4 DPI and 10 DPI and clinically evaluated for 28 days. Recovered ticks were reared to the next developmental stage and/or tested by qPCR for detection of rickettsial DNA. Molted, unfed ticks were allowed to feed on tick-naïve rabbits and guinea pigs, which were clinically evaluated for 21 days and tested by seroconversion by testing paired serum samples (days 0 and 21 post-infestation) against *R. rickettsii* antigens

At day 0 (zero) of the study, chamber A of wild boars 1–4 (G1) received 45 *A. sculptum* adults (26 males and 19 females) derived from the *R. rickettsii*-infected cohort. Wild boar no. 5 (G2) was infested with 60 *A. sculptum* non-infected adults (30 males and 30 females) derived from an uninfected cohort. Four days after infestation (DPI) with infected ticks, chamber B of G1 wild boars and the single chamber of G2 wild boar received approximately 1000 larvae and 1000 nymphs from the uninfected cohort of *A. sculptum*; this procedure was repeated on 10 DPI (Fig. 1).

From 0 to 28 DPI, all five wild boars were examined daily to monitor their health status and rectal temperature. A wild boar was considered febrile if rectal temperature was > 40 ºC for at least 2 consecutive days [38, 39]. Blood samples (3.0 ml in EDTA) were collected from each animal through the cephalic vein every 3 days from 0 to 28 DPI. Each blood sample was divided into three sub-aliquots to be used: at hematological tests, at DNA extraction and to obtain plasma for serological testing. At 6, 9, 12 and 15 DPI, additional blood samples were collected and used for inoculation into guinea pigs (techniques described below). From the control group (G2), a single additional blood collection at 9 DPI was used for inoculation of guinea pigs.

Before each procedure with wild boars (sample collections and tick feeding chamber preparation), animals were sedated with a mixture of xylazine (2 mg/kg) and ketamine (20 mg/kg) by the intramuscular route (IM) [40]. At the end of the experiment, the wild boars were euthanized with an overdose of the combination of ketamine and xylazine (IM), followed by potassium chloride solution, by intracardiac injection [41].

### Guinea pig inoculation

**Fig. 2:**
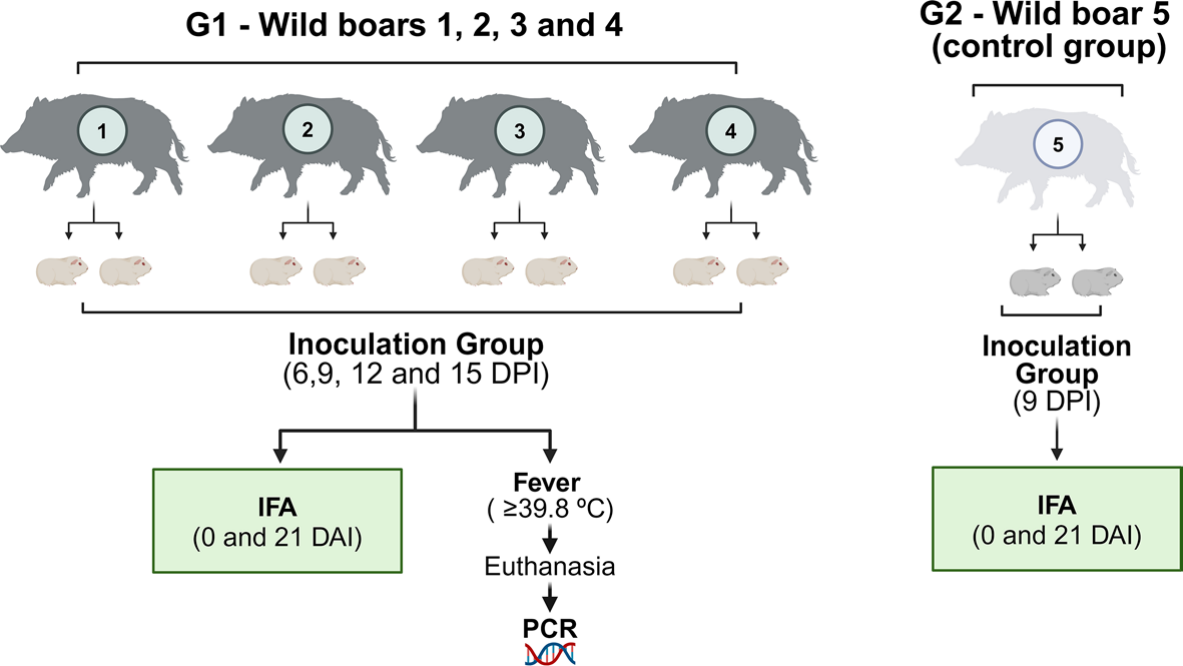
Scheme of guinea pig inoculation. For each wild boar from infected group, two guinea pigs were simultaneously allocated for intraperitoneal inoculation with 1.0 ml of anticoagulated blood collected at 6, 9, 12 and 15 DPI; for the animals from control group, two guinea pigs were used for inoculation of anticoagulated blood collected at 9 DPI. The inoculated guinea pigs were clinically evaluated during 21 days and tested by seroconversion by testing paired serum samples (days 0 and 21 DAI) against *R. rickettsii* antigens. *DAI* days after inoculation

To identify a probable bacteremia due to *R. rickettsii* in wild boars [16, 42] from the infected group, two guinea pigs were simultaneously allocated for intraperitoneal inoculation with 1.0 ml of anticoagulated blood collected from each of wild boars 1 to 4 (infected group) at 6, 9, 12 and 15 DPI. For wild boar 5 (control group), this procedure was performed only at 9 DPI (Fig. 2). For this purpose, guinea pigs had been previously anesthetized with a mixture of xylazine (5 mg/kg) and ketamine (25 mg/kg) (IM) [43], and a blood sample was collected by intracardiac puncture for IFA analyses (day 0) against *R. rickettsii* [36, 37]. The clinical signs and rectal temperature of all guinea pigs were monitored daily for 21 days. A second blood sample was collected at day 21 (days after inoculation) for IFA analyses, and thereafter guinea pigs were killed with an overdose of the combination of ketamine and xylazine (IM) followed by potassium chloride solution by intracardiac injection [41].

Until the time of inoculation, guinea pigs were clinically healthy. If fever was detected, the guinea pig was euthanized for collection of spleen and lung for DNA extraction followed by qPCR [34] (descriptions of fever parameters and qPCR assay are described below).

### Evaluation of tick acquisition and transmission of rickettsiae

The engorged larvae, nymphs and females that naturally detached from wild boar skin inside the feeding chambers were collected daily and transferred to an incubator at 27 ± 1 °C and 80 ± 5% relative humidity, where they were observed for ecdysis or complete oviposition. Unfed nymphs and adults, between 2 and 4 weeks post molting and corresponding to different days of detachment from the wild boars, were subjected to DNA extraction and qPCR [34] to verify the percentage of ticks potentially infected with *R. rickettsii* (in the case of those from G1) or to confirm the absence of infection in ticks fed on the G2 wild boar.

To assess the competence of wild boars to transmit *R. rickettsii* through tick-acquisition feeding, nymphs and adults (molted from larvae and nymphs, respectively, after feeding on wild boars) were used to infest tick-naïve guinea pigs and rabbits, respectively. Two rabbits and two guinea pigs were used for ticks that fed on each G1 wild boar. Guinea pigs received nymphs derived from engorged larvae that detached from wild boars up to 17 DPI, whereas rabbits received adults derived from engorged nymphs that detached from 9 to 18 DPI (Fig. 1). Infestations consisted of 200–450 nymphs per guinea pig and 74–96 adult ticks per rabbit (37–45 males plus 37–51 females), depending on the availability of ticks. These ticks were released inside feeding chambers that had been previously glued to the shaved back of the host, as described [44]. The chambers were checked daily for the presence of naturally detached engorged ticks, which were collected and maintained in the same above-mentioned incubator until ecdysis or complete oviposition.

Blood samples were collected at 0 and 21 DPI from rabbit and guinea pigs by saphenous vein and intracardiac puncture, respectively, to verify the occurrence of seroconversion to *R. rickettsii*. Before tick infestation, guinea pigs and rabbits underwent clinical evaluation and were seronegative (serum dilution of 1:64) by IFA against crude antigens of *R. rickettsii*. The fixation of the infestation chambers and the collection of blood from the animals were performed under anesthesia (xylazine 5 mg/kg + ketamine 25 mg/kg, IM) [40, 43]. Plasma was separated by centrifugation (5000×*g* for 10 min) and stored at − 20 °C until tested by IFA (described below). Guinea pigs were considered febrile if rectal temperature was ≥ 39.8 °C for at least 3 consecutive days, and rabbits were considered febrile if rectal temperature was > 40 ºC for at least 2 consecutive days [42, 45]. If fever was detected, the animal was euthanized for collection of spleen and lung for DNA extraction followed by qPCR assay [34].

### Hematology tests

Whole blood samples from wild boars were used for estimation of packed cell volume (PCV) by the microhematocrit technique and for estimation platelet, following the procedures described by Katsogiannou et al. [46]. The values were compared with previously reported reference values for domestic pigs because there are no standardized values for wild boars [38, 39].

### Serological analyses (IFA)

Blood samples collected in tubes with EDTA were centrifuged at 5000×*g* for 10 min, and the separated plasma was kept at − 20 ^◦^C until processing. Plasma from wild boars, guinea pigs and rabbits was tested by IFA using *R. rickettsii* (strain Pampulha) crude antigen [13]. Wild boar samples were also tested for IFA using crude antigens fixed on slides, derived from three other isolates of *Rickettsia* spp. from Brazil: *R. parkeri* strain At24 [33], *R. bellii* strain Mogi [35] and *R. amblyommatis* strain Ac37 [47].

Plasma samples were tested individually using the methodology described by Horta et al. [36]. However, unlike Horta et al. [36], we used plasma instead of serum to detect IgG antibodies. Previous studies showed that there was no difference between serum and plasma samples for antibody detection [48, 49]. Briefly, the plasma was diluted in two-fold increments with phosphate-buffered saline (PBS), starting from the 1:64 dilution. Slides were incubated with rabbit anti-pig IgG (whole molecule, IgG, Sigma Diagnostics), goat anti-rabbit IgG (Sigma, St Louis, MO, USA) and rabbit anti-guinea pig IgG (Sigma, St Louis, MO, USA), coupled with fluorescein isothiocyanate at 1:400 dilution for wild boars, at 1:500 dilution for rabbit and at 1:100 dilution for guinea pigs. Samples were initially tested in a 1:64 dilution (with PBS) as cut-off, and those that were reactive were further diluted in twofold increments to the endpoint titer, as reported earlier [36]. Each slide included a previously known reactive serum (positive control) and non-reactive serum (negative control) at 1:64 dilution for each animal species (wild boars, rabbits or guinea pig).

### Molecular analyses

Ticks were processed individually for DNA extraction by the guanidine isothiocyanate protocol [50]. Blood DNA was extracted using the DNeasy Blood & Tissue Kit (Qiagen, Valencia, CA, USA), following the manufacturer's protocols. Final products were stored at − 20 °C for further amplification by polymerase chain reaction (PCR).

DNA samples were initially tested by a real-time PCR assay (TaqMan qPCR) that amplifies a 147-bp fragment of the citrate synthase gene (*glt*A) of *Rickettsia* spp. by using the primers CS-5 (forward) and CS-6 (reverse) and an internal fluorogenic probe (6-FAM d, BHQ-1) (Integrated DNA Technologies, San Diego, CA), according to a previously reported protocol [34, 51]. The sensitivity of the technique was determined to be one DNA copy of *R. rickettsii* [34]. Each PCR run included positive (DNA of *Rickettsia vini*-infected in Vero cells) and negative (molecular-grade water) controls [34].

DNA samples considered positive by the *glt*A qPCR protocol were further tested by a conventional PCR (cPCR) targeting a 632-bp fragment of the 190-kDa outer membrane protein gene (*omp*A), using the primers Rr190.70 [52] and Rr190.701 [53]. Each reaction included positive and negative control samples, as mentioned above. In the Results section, samples containing rickettsial DNA were those that yielded PCR-positive results by both qPCR (cycle threshold < 35) and *omp*A-conventional PCR.

Fed adult ticks recovered from chamber A of the G1 wild boars were also tested by the qPCR protocol for *Rickettsia* spp. In this case, the positive samples were tested by a second qPCR assay targeting a gene encoding the hypothetical protein A1G_04230 of *R. rickettsii* (GenBank accession no. ABV76353; *R. rickettsii* Sheila Smith strain), using the primers RRi6_F (forward) and RRi6_R (reverse) and an internal fluorogenic probe (Fl-TCC TCT CCA ATC AGC GAT TC, BHQ-1) [54]. This protocol was performed to confirm that G1 wild boars were exposed to *R. rickettsii*-infected adults.

As quality control for probable amplification inhibition and for the DNA extraction method, all samples that tested negative for rickettsial DNA by the qPCR were retested by two cPCR. Tick samples were tested by cPCR targeting the tick mitochondrial *16S* rDNA gene [55], while blood samples were tested to amplify the mammal mitochondrial cytochrome b gene (*cyt*B) [56]. If a DNA sample did not yield products of the expected size in this cPCR assay, it was discarded from the study. Conventional PCR products for the *16S* rDNA*, omp*A and *cyt*B genes were stained with SYBR Safe (Invitrogen, Carlsbad, CA, EUA), following the manufacturer's protocols, and visualized by electrophoresis in a 1.5% agarose gel using an ultraviolet transilluminator.

## Results

### Wild boars clinical aspects

**Fig. 3:**
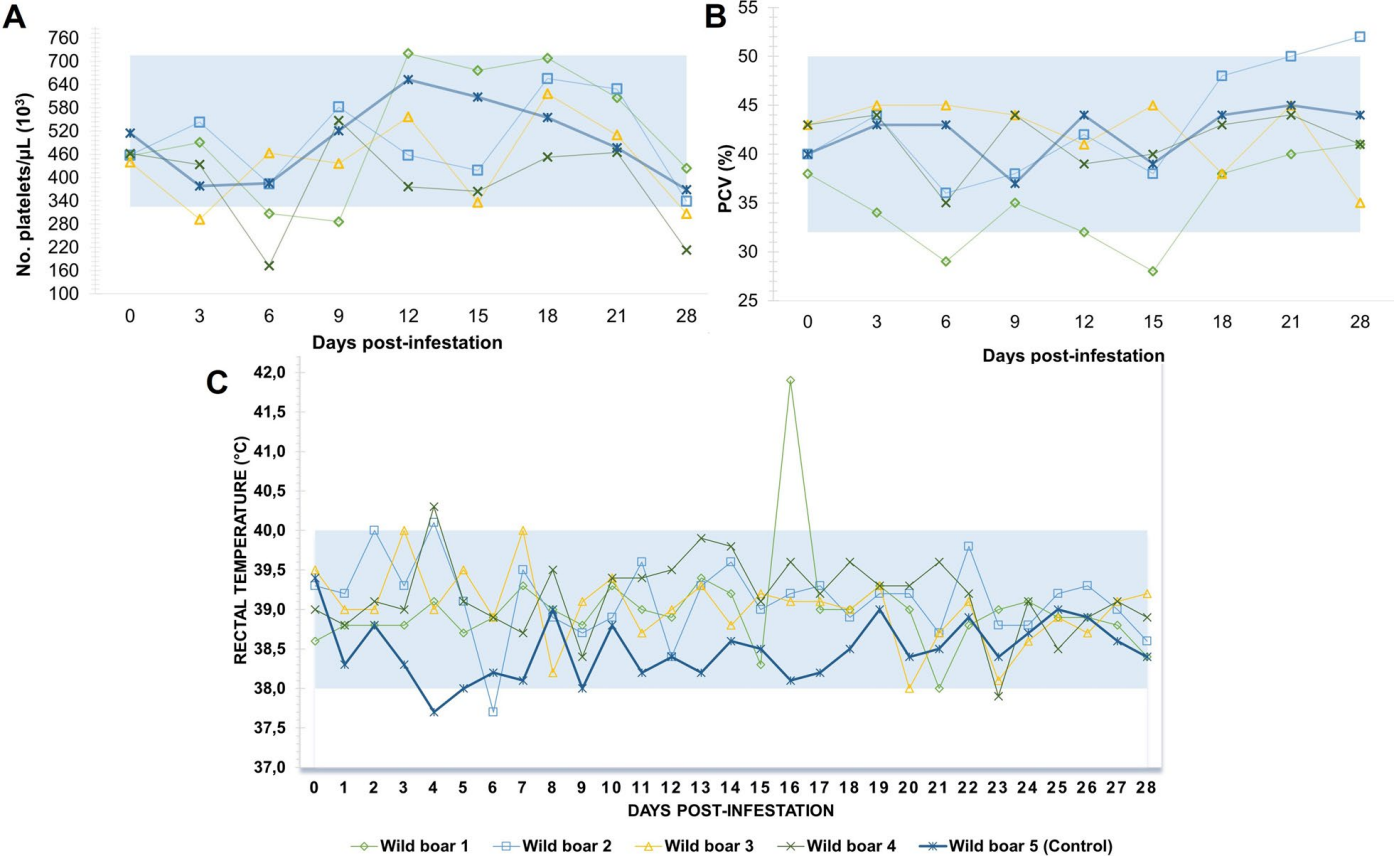
Platelets estimative (**A**), packed cell volume (PCV) (**B**) and rectal temperature (**C**) of four wild boars (nos. 1–4) experimentally infected with *Rickettsia rickettsii* and one uninfected wild boar (no. 5) during 28-day monitoring. Blue areas represent the reference ranges for domestic pigs (*Sus scrofa*) [38, 39]

None of the five wild boars showed consistent clinical changes during the 28 days of the experiment. In general, fever was not detected in the wild boars infected with *R. rickettsii* and in the control group, as the highest rectal temperature values (> 40.0 °C) did not last for > 1 consecutive day. Wild boar no. 1 showed a slight increase (41.9 °C) at 16 DPI, decreasing to 39.0 °C the following day. Hematocrit, estimated platelet count and temperature values of the wild boars are shown in Fig. 3. Some laboratory measurements were slightly different from reference values, but without clinical relevance, as they were observed on sparse days. Although wild boar nos. 1, 2 and 4 showed a downward trend in hematological values (platelet count and PCV) at 6 DPI, these values remained within the reference range or close to the minimum values (Fig. 3).

### Serology

Before infestation (0 DPI), wild boar no. 5 (G2 control group) was seronegative for *R. rickettsii* in the IFA, but anti-*R. parkeri* antibodies were detected with the marginal endpoint titer of 64. Throughout the study, this animal remained seronegative for *R. rickettsii, R. amblyommatis* and *R. bellii* and did not show any alteration of the 64-endpoint titer to *R. parkeri*.

**Fig. 4:**
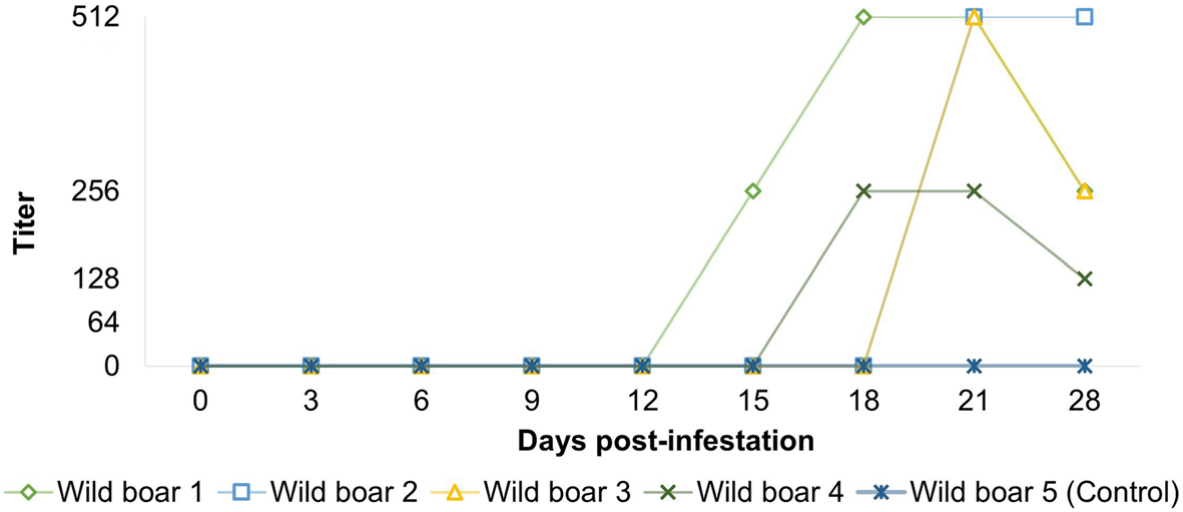
Antibody titers (IFA ≥ 64) in wild boars experimentally infected with *Rickettsia rickettsii*. *IFA* indirect immunofluorescence assay

On 0 DPI, the four wild boars of G1 (nos. 1–4) were seronegative for *Rickettsia* spp. (*R. rickettsii, R. parkeri, R. amblyommatis* and *R. bellii*). Anti-*R. rickettsii* antibodies (titers ≥ 64) were first detected at 15 DPI (wild boar no.1, titer 256), 18 DPI (wild boar no. 4, titer 256) and 21 DPI (wild boars no. 2 and 3, titer 512). At 21 DPI, the four G1 animals presented anti-*R. rickettsii* antibodies that remained stable or decreased by one endpoint titer at the 28 DPI. The highest titers were 512 for wild boar nos. 1, 2 and 3 and 256 for wild boar no. 4 (Fig. 4).

Comparative tests between antigens showed that all *R. rickettsii*-positive samples were also positive for *R. parkeri*, while some of these samples were also positive for the remaining two antigens. Titers for *R. rickettsii* were higher than or equal to those for other antigens (Table 1).

**Table 1:** IFA endpoint titers for four *Rickettsia* species in wild boars experimentally infected with *Rickettsia rickettsii*

Wild boar no.	DPI^a^	IFA titers for *Rickettsia* antigens			
		*Rickettsia rickettsii*	*Rickettsia parkeri*	*Rickettsia amblyommatis*	*Rickettsia bellii*
1	0	–	–	–	–
	15	256	256	–	–
	18	512	512	128	256
	21	512	512	256	512
	28	256	512	128	256
2	0	–	–	–	–
	21	512	256	128	128
	28	512	256	128	128
3	0	–	–	–	–
	21	512	512	256	128
	28	256	256	128	64
4	0	–	–	–	–
	18	256	256	–	–
	21	256	256	256	256
	28	128	128	128	128

(–): nonreactive at titer ≥ 64

^a^Except for samples collected from animals 0 DPI, we only included results from days in which the animals showed seroreactivity for any *Rickettsia* sp. antigens tested

### Search for *Rickettsia* in wild boar blood

Blood samples from the four wild boars of G1 were negative according to qPCR throughout the evaluating period. None of the guinea pigs inoculated with wild boar blood showed hyperthermia or clinical signs compatible with *R. rickettsii* infection. However, at 21 days after inoculation (DAI), seroconversion with low endpoint titers (64–256) was observed in several guinea pigs after being inoculated with blood from the G1 wild boars (Table 2). This included two guinea pigs inoculated with blood collected from wild boar nos. 3 and 4 on 9 DPI, one guinea pig inoculated with blood from wild boar no. 3 on 12 DPI and seven guinea pigs inoculated with blood from wild boar 1–4 on 15 DPI.

**Table 2:** IFA endpoint titers in guinea pigs inoculated with wild boar blood

DPI	Wild boar no. 1		Wild boar no. 2		Wild boar no. 3		Wild boar no. 4	
	Guinea pigs inoculated no.	IFA	Guinea pigs inoculated no.	IFA	Guinea pigs inoculated no.	IFA	Guinea pigs inoculated no.	IFA
6	1	–	3	–	5	–	7	–
	2	–	4	–	6	–	8	–
9	9	–	11	–	13	128	15	–
	10	–	12	–	14	–	16	128
12	17	–	19	–	21	128	23	–
	18	–	20	–	22	–	24	–
15	25	256	27	256	29	256	31	–
	26	128	28	64	30	256	32	128^a^

(−): nonreactive at titer ≥ 64

*IFA* indirect immunofluorescence assay, *DPI* days post-infestation with infected tick colony

^a^Guinea pig showed titer 1024 before inoculation

Before inoculation with wild boar blood (0 DAI), all guinea pigs were seronegative (titer < 64) to *R. rickettsii*. The only exception was guinea pig no. 32 (inoculated with blood from wild boar no. 4 collected on 15 DPI), which presented an endpoint titer  of 1024 at 0 DAI and then an endpoint titer of 128 at 21 DAI. The two guinea pigs inoculated with blood from wild boar no. 5 (G2) on 9 DPI remained seronegative at 21 DAI.

### Tick acquisition and transmission of rickettsiae

Regarding unfed nymphs and adults that fed on wild boars as larvae and nymphs, respectively, none revealed rickettsial DNA by qPCR (Table 3). However, one unfed adult tick recovered from the animal in the control group was positive in the qPCR targeting the rickettsial *glt*A gene. Nonetheless, this sample was negative in cPCR for the *omp*A gene of SFG rickettsiae. Among adult ticks from the infected colony that had fed on G1 wild boars (recovered from feeding chamber A), the qPCR positivity for *R. rickettsii* was 33.3% (3/9), 33.3% (2/6), 25.0% (2/8) and 10.0% (1/10), recovered from wild boar nos. 1, 2, 3 and 4, respectively (Table 3).

**Table 3:** Results of qPCR assay for *Rickettsia* spp. on ticks that fed on wild boar no. 1 to 4 (infected group) and no. 5 (control group)

Wild boar no.	No. of positive ticks in real-time PCR/no. of tested ticks (%)		
	Unfed nymphs (fed as larvae on wild boars)	Unfed adults (fed as nymphs on wild boars)	Adult ticks^b^
1	0/45 (0)	0/57 (0)	3/9 (33.3)
2	0/45 (0)	0/75 (0)	2/6 (33.3)
3	0/40 (0)	0/41 (0)	2/8 (25)
4	0/55 (0)	0/52 (0)	1/10 (10)
5 (control)	0/33 (0)	1/45 (2.2)^a^	0/7 (0)
**Total**	**0/218 (0)**	**1/270 (0.4)**	**8/40 (20)**

^a^The sample was negative for the gene of the outer membrane protein (*omp*A) present in SFG rickettsiae in cPCR

^b^Refers to adult ticks that fed on wild boar nos. 1–4 derived from the *R. rickettsii*-infected colony, whereas the adult ticks that fed on wild boar no. 5 were derived from the uninfected colony

Wild boar-derived ticks that successfully fed on rabbits and guinea pigs consisted, respectively, of 141 adults and 349 nymphs from wild boar no. 1, 163 adults and 217 nymphs from wild boar no. 2, 164 adults and 270 nymphs from wild boar no. 3 and 169 adults and 271 nymphs from wild boar no. 4. None of the infested rabbits and guinea pigs seroconverted to *R. rickettsii* or presented any clinical signs compatible with rickettsiosis (Table 4). Only one rabbit became febrile, but between 10 and 13 DPI, the period corresponding to days with the highest number of engorged females detachment from the chamber, and the animal appeared moderately more irritable. Therefore, no animal was euthanized before 21 days post-infestation for research on rickettsial DNA in organs.

**Table 4:** Infestations of naïve rabbits and guinea pigs with unfed adults and nymphs that had molted from engorged nymphs and larvae, respectively, after feeding on wild boars

Wild boar no.	Rabbit no. infested with adults			Guinea pig no. infested with nymphs		
	Rabbits	Fever^a^	IFA	Guinea pigs	Fever^a^	IFA
1	COE 1	No	n.r	COB 1	No	n.r
	COE 2	Yes^b^	n.r	COB 2	No	n.r
2	COE 3	No	n.r	COB 3	No	n.r
	COE 4	No	n.r	COB 4	No	n.r
3	COE 5	No	n.r	COB 5	No	n.r
	COE 6	No	n.r	COB 6	No	n.r
4	COE 7	No	n.r	COB 7	No	n.r
	COE 8	No	n.r	COB 8	No	n.r

^a^Rabbits and guinea pigs were considered to have fever if rectal temperature was > 40.0 °C and ≥ 39.8 ºC for at least 2 and 3 consecutive days between 0 and 21 days after tick infestation, respectively

^b^Rectal temperatures were 40.4 °C at 10 DPI and 40.1 °C at 11 DPI to 13 DPI

*n.r.* nonreactive at titer ≥ 64, *IFA* indirect immunofluorescence assay, *DPI* days post-infestation

## Discussion

This was the first study to perform experimental infection of wild boars (*S. scrofa*) by *R. rickettsii* via tick infestation, simulating natural conditions. Moreover, the role of these animals as amplifying host for *A. sculptum* ticks was evaluated under laboratory conditions. Our results showed that all wild boars seroconverted after being infested by *A. sculptum* infected by *R. rickettsii*. However, they did not show signs of fever or consistent hematological abnormalities, in contrast to other mammal species (humans, capybaras, dogs, guinea pigs and domestic rabbits), in which *R. rickettsii* usually induces high fever, vascular abnormalities like edema, erythema and/or necrosis, and even death [42, 57–59]. Except for wild boar no. 1, in the infected group, the highest rectal temperature observed was 40.3 °C (wild boar no. 4), and the highest values (> 40.0 ºC) did not remain for 2 consecutive days. In addition, the temperature elevation generally occurs as an effect of agitation and stress, especially when dealing with animals not accustomed to daily handling [39, 60], which was observed in the animals in this experiment.

Wild boar no. 1 presented a temperature of 41.9 ºC on 16 DPI and presented slightly lower PCV values and higher platelet estimates, the latter mainly between 12 and 21 DPI. This period of the experiment coincided with a clinical condition observed on 12 DPI, where the animal presented a purulent lesion in the inguinal region, the site of the castration wound, suggesting a complication caused by this surgical procedure that had been performed some weeks before the study. This would justify the punctual increase in temperature and the change in platelet count and PCV values, since inflammatory processes can cause changes in temperature and hematological values, such as an increase in the number of platelets [38, 39]. The other male wild boar in this study (no. 2) did not show any clinical alteration in its castration site.

Our results from inoculation of guinea pigs with wild boar blood provided evidence that wild boar did not develop efficient bacteremia during the study period, since their blood did not cause disease in the guinea pigs, although we detected seroconversion of guinea pigs that were inoculated with blood collected 9, 12 and 15 days after infestation of wild boars with *R. rickettsii*-infected ticks. In previous studies involving experimental infestation of capybaras and opossums (*Didelphis aurita*) with *R. rickettsii*-infected *A. sculptum* ticks, bacteremia was convincingly demonstrated because inoculation of guinea pigs with capybara or opossum blood (just like done in the present study with wild boar blood) was able to cause hyperthermia and other clinical signs in guinea pigs, which in turn presented seroconversion with high antibody titers [16, 42].

Ramírez-Hernández et al. detected *R. rickettsii*-bacteremic periods in capybaras between 6 and 16 DPI when more than half of the blood-inoculated guinea pigs presented fever and vascular abnormalities such as edema, erythema and/or necrosis, seroconverting to final titers ≥ 131,072 [42]. In the present study, the highest titers of the blood-inoculated guinea pigs were 256 (titers range 64–256), suggesting that the bacterial load present in the blood of wild boars was insufficient to cause infection, since guinea pigs are extremely susceptible to *R. rickettsii* infection [42, 61]. A previous laboratory study demonstrated that a minimum inoculation dose of 21 to 126 organisms of *R. rickettsii* is sufficient to induce clinical infection with seroconversion in guinea pigs [62]. Hence, it is possible that the guinea pigs that seroconverted with low endpoint titers were not exposed to viable *R. rickettsii* organisms, or if they were, it could have been < 21 viable organisms. This result indicates a minimal rickettsial load in wild boar blood during the study, which corroborates our negative results for the molecular detection of rickettsial DNA in wild boar blood and postmolted acquisition ticks.

Unfortunately, we did not continue inoculating guinea pigs with wild boar blood collected after 15 DPI to determine whether subsequent groups of guinea pigs would continue to seroconvert or develop fever and/or other clinical signs. However, according to the literature, no other animal species developed bacteremia because of *R. rickettsii* after 9 DPI, except that reported for opossum *D. aurita*, which showed an intermittent pattern of bacteremia [63]. Furthermore, rickettsial DNA was not detected in the blood of wild boars; however, this was expected since it is known that *Rickettsia* spp. multiplies inside the endothelial cells of vertebrates; therefore, its concentration in the blood is at low levels and usually cannot be detected by molecular analysis [45].

Among the acquired ticks that fed on wild boars of G1 (from Chamber B), none tested positive for *Rickettsia* by qPCR, and the absence of disease or seroconversion in rabbits and guinea pigs infested with these ticks indicates that rickettsial infection was not established in the ticks. That is, wild boars did not develop bacteremia of sufficient magnitude to infect *A. sculptum*, and these arthropods did not transmit the bacteria to the next host. These results indicate that wild boars are not capable of acting as amplifying hosts for *A. sculptum* larvae and nymphs, which would result in efficient transmission by feeding on the subsequent developmental stage (nymphs and adults, respectively).

All infected wild boars developed a humoral immune response, although they presented moderate antibody titers, especially anti-*R. rickettsii* antibodies, with endpoint titers of 256 or 512. Previous studies had already reported seropositivity for *Rickettsia* spp. in wild boars under natural conditions, especially in areas where *A. sculptum* was the most prevalent tick species [21–23]. In those studies, despite the high seropositivity for *Rickettsia* spp. (72.2–77.4%), the animals presented anti-*R. rickettsii* endpoint titers between 512 and 1024, which were usually similar (less than four-fold difference) from the other *Rickettsia* species. Similarly, in the present study, wild boars presented moderate endpoint titers for *R. rickettsii* that cross-reacted with three other *Rickettsia* species, usually with similar or less than four-fold difference between each other.

Our study demonstrated that wild boars did not present a notable humoral response, which might not be persistent because it started to drop in most of the animals by 30 days after infection. In contrast, experimental infection of horses with *R. rickettsii* showed anti-*R. rickettsii* antibodies for the first time at 12 DPI (titers 256), peaking between 18 and 20 DPI (titers range 2148–8192) and persisting for at least 2 years when the IgG antibody titer was 256 [61]. In our study, wild boars first presented anti-*R. rickettsii* antibodies between 15 and 21 DPI, reaching a peak between 18 and 21 DPI (titers 512). Although we performed serological monitoring of wild boars for only 28 days, the results demonstrate that from 28 DPI onwards, antibodies showed a decreasing trend, and future studies are needed to evaluate the persistence of IgG antibodies in wild boars after *R. rickettsii* infection. Interestingly, both horses [61] and wild boars (present study) did not develop bacteremia due *R. rickettsii* and cannot be considered amplifying hosts of this bacterium for *A. sculptum* ticks. However, it seems that horses would be better sentinels than wild boars for BSF surveillance based on serological analyses. This statement needs to be further evaluated by sampling wild boars from BSF-endemic areas, similar to what has been done with horses [36, 50].

The present protocol for producing the *R. rickettsii-*infected colony was exactly the same as reported in previous studies in our laboratory [11]. In all cases, the same lineage of *R. rickettsii* (strain Itu) was used, and we performed experimental infection of *A. sculptum* by infesting larvae and nymphs on guinea pigs that were previously inoculated with infected guinea pig organs. This procedure has been previously performed with five independent colonies of *A. sculptum,* each from a distinct geographical site in Brazil (including areas endemic and nonendemic for BSF). In all cases, it resulted in some *R. rickettsii-*infected ticks, which were demonstrated to be competent vectors of *R. rickettsii* to susceptible hosts [11]. These results validate the present experimental infection protocol using *A. sculptum* ticks infected with *R. rickettsii*, which reflects a natural condition much more than if we had parenterally inoculated wild boars with *R. rickettsii*.

Adult ticks used to infest G1 wild boars (infected group) derived from a tick colony that demonstrated a 30% infection rate. Furthermore, the adult ticks from this infected colony, tested by PCR after they had fed on wild boars, demonstrated infection rates varying from 10 to 33.3%. Thus, considering that each wild boar in the infected group (G1) was infested with 45 ticks from the infected colony, which remained on the animals for up to 21 days (the day on which all ticks that were still attached were removed), it can be inferred that wild boars received considerable amounts of bacteria through tick feeding, with each wild boar receiving at least 4–15 *R. rickettsii-*infected adult ticks. Notably, < 1% of the *A. sculptum* population is infected by *R. rickettsii* within the BSF-endemic areas [6, 10, 12, 14]. Hence, in our experiment, we simulated an infection by *R. rickettsii* via infestation by a tick population presenting an infection rate at least 10 times higher than what is found under natural conditions. These data reinforce our results, i.e. even when exposed to a relatively high amount of *R. rickettsii-*infected *A. sculptum* ticks, wild boars did not develop clinical disease and did not serve as rickettsial amplifying hosts for *A. sculptum* ticks.

Despite the present results, wild boars have been shown to be suitable hosts for *A. sculptum* ticks; therefore, their importance in the epidemiology of BSF cannot be ruled out. Due to their ability to travel long distances and to be found in different ecosystems, wild boars can carry and disperse *A. sculptum* ticks from their original habitats to other ecosystems [22, 26–28]. Thus, wild boars can potentially lead to the synergistic dissemination of *A. sculptum*, especially to regions where there are other hosts such as capybaras, thus contributing to the greater risk of human exposure to the tick vector and consequently to *R. rickettsii*.

## Conclusions

Our results indicate that wild boars cannot transmit *R. rickettsii* to *A. sculptum* ticks and that probably they are not relevant in the maintenance cycle of this bacterium in nature. However, future studies are essential to better assess the impact that these animals can cause, especially in the dispersion of potentially infected ticks between different regions of the country, mainly in biomes where there are already known amplifying hosts of *R. rickettsii*.

## Data Availability

No datasets were generated or analyzed during the current study.

## Abbreviations

BSF: Brazilian spotted fever
CONCEA: Brazilian National Council of Animal Experimentation
cPCR: Conventional polymerase chain reaction
*cyt*B: Mammal mitochondrion cytochrome *b* gene
DAI: Days after-inoculation
DNA: Deoxyribonucleic acid
DPI: Days post-infestation
EDTA: Ethylenediaminetetraacetic acid
*glt*A: Citrate synthase enzyme encoding gene
IFA: Imunofluorescence assay
IgG: Immunoglobulin G
IM: Intramuscular
*omp*A: Outer membrane protein A gene
PBS: Phosphate-buffered saline
PCR: Polymerase chain reaction
PCV: Packed cell volume
qPCR: Real-time quantitative polymerase chain reaction
SFG: Spotted fever group
